# Immunomodulatory effects of photothermal therapy in breast cancer: advances and challenges

**DOI:** 10.3389/fimmu.2025.1544693

**Published:** 2025-07-04

**Authors:** Lin Chen, Hanyu Zhang, Yi Hou, Wang Yunlong, Xianhu Feng

**Affiliations:** ^1^ Nanchong Key Laboratory of Individualized Drug Therapy, Department of Pharmacy, Beijing Anzhen Nanchong Hospital Capital Medical University & Nanchong Central Hospital, The Second Clinical Medical College, North Sichuan Medical College, Nanchong, Sichuan, China; ^2^ Clinical Pharmacy and Pharmacy Department of The People’s Hospital of Zhongjiang, Deyang, Sichuan, China

**Keywords:** breast cancer, photothermal therapy, immunogenic cell death, immune response, the tumor microenvironment

## Abstract

Breast cancer (BC) is the leading cause of cancer death in women, partly because of the significant toxicity and low specificity associated with chemotherapy drugs. Photothermal therapy (PTT) provides a new paradigm for the precise treatment of BC through local thermal ablation and immune regulation. PTT utilizes the heat generated by laser irradiation to kill tumor cells. Notably, PTT can activate the innate and adaptive immune systems by releasing antigens, which are then presented by antigen-presenting cells (APCs). These antigens are primarily released through various forms of tumor cell death induced by the thermal effects of PTT. The process of PTT-activated anti-immunity involves T cells, dendritic cells (DCs), B cells, natural killer (NK) cells, and macrophages. Therefore, regulation of the immune system by PTT in BC is considered as a promising therapeutic approach. This review elucidates the mechanisms by which PTT regulates anti-tumor immune responses through processes such as antigen release, antigen presentation, and immune cell activation. We also focus on the latest advancements and challenges in nanomaterials research, preclinical studies, and translational trials for PTT in BC treatment. This review is expected to improve our understanding of the anti-tumor effects of PTT based on the immune cycle of BC. It is expected to address critical gaps in PTT-based immunotherapy for BC, such as insufficient antigen release, the immunosuppressive microenvironment, and the transformation of cold tumors.

## Graphical Abstract

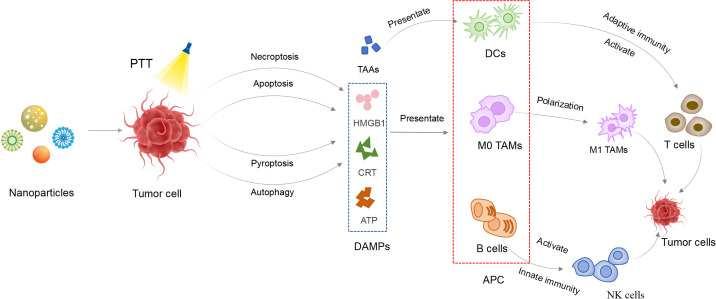

## 1 Introduction

BC is the most prevalent type of cancer among women and is the primary factor contributing to cancer-related fatalities in women globally, exhibiting a mortality rate of 6.9% (1). In 2025, approximately 319,750 women in the United States are estimated to develop BC and 42,680 will die from it (2). Genetic factors, demographic structure, lifestyle, and environmental elements may influence the incidence, survival rate, and mortality of the disease (3). Various factors contribute to the high heterogeneity of BC among individual patients with different clinical manifestations. Tumor heterogeneity leads to diverse molecular characteristics, including activation of human epidermal growth factor receptor 2 (HER2), hormone receptor (HR) activation, and/or BRCA mutations (4). These characteristics distinguish BC from other malignant tumors according to tumor metastasis and drug resistance. Studies have found that the elevated expression of Hsp27, Hsp60, Hsp70, and Hsp90 in BC tissues is closely associated with tumor aggressiveness, epithelial-mesenchymal transformation (EMT), metastasis, and treatment resistance (5, 6). Tumor metastasis is the primary cause of mortality in BC patients, in which breast cancer stem cells (BCSCs) play a critical role (7). For various types of BC, treatment involves multidisciplinary and multilayered strategies, including endocrine therapy, radiation therapy, chemotherapy. However, these therapies face significant challenges, including limited efficacy, drug resistance, and harmful side effects, necessitating the development of new treatment strategies that are both precise and safe.

In recent years, PTT has garnered widespread attention as an effective and significant strategy. Compared to traditional treatment methods, PTT offers advantages such as high selectivity, non-drug resistance, and minimal invasiveness (8). The strategy of directly delivering PTT to breast ducts through microcatheters has been proven to be clinically feasible (9, 10). PTT relies on the conversion of light energy into thermal energy (11). This process is primarily achieved through photothermal agents (PTAs), such as organic materials, organic frameworks, noble metal nanostructures, and carbon-based nanomaterials (12–14). Near infrared (NIR) utilized in PTT can penetrate only to a depth of 1–2 cm below the body surface, providing treatment conditions for superficial malignant tumors such as BC (11). Research has found that cells die when the temperature rises and damages DNA beyond a certain threshold (15). Notably, tumor tissues with vascular malformations are more sensitive to thermal stimulation compared to normal tissues, which serves as the primary basis for PTT in tumor treatment (16). The latest research has found that light-induced hyperthermia can damage the integrity of cell membranes, leading to chemical damage caused by the influx of Ca^2+^ (17). Additionally, PTT has been found to cause additional damage to the vascular system supplying the tumor (18). However, when tissue temperature rises to 41°C, the body initiates a heat shock response, producing heat shock proteins (HSPs) to counteract the initial thermal damage effects (19). Research has found that high temperatures not only induce necrosis of tumor tissues but also affect the microenvironment of tumor cells, triggering programmed cell death (PCD) (20, 21). Intracellular hyperthermia has been proven to stimulate the release of tumor antigens and pro-inflammatory cytokines, including heat HSPs, adenosine triphosphate (ATP), and high mobility group protein 1 (HMGB1) (22, 23). Recent studies have shown that PTT induces the release of damage-associated molecular patterns (DAMPs), activating anti-tumor immune responses (such as DCs maturation and CD8+ T cells infiltration), providing new insights into inhibiting BC metastasis (20, 21, 24). Furthermore, the immune effects of PTT on BC have been confirmed through preclinical trials. This study systematically reviews the mechanisms of PTT in BC, particularly its impact on immune cells such as T cells, DCs, B cells, macrophages, and NK cells. Additionally, clinical trials involving PTT in the treatment of BC are discussed. This study aims to lay the foundation for future immunotherapy in BC, guiding personalized treatment for the disease.

## 2 Nanoparticles

PTAs absorb photon energy and transition from the singlet ground state to the excited singlet state under irradiation from external light sources such as NIR. The excess energy is dissipated through nonradiative vibrational relaxation, leading to a temperature increase in the surrounding microenvironment and generating a thermal effect (11). Researchers have made significant progress in the field of PTT for tumors such as gastric cancer, liver cancer, and BC by utilizing materials with photothermal properties (25, 26). These materials exhibit excellent anti-cancer effects in the near-infrared region. And the imaging based on specific PTAs can greatly enhance the diagnostic accuracy and treatment efficiency of cancer. The success of PPT largely depends on the selection of PTAs. Various types of photothermal nanotherapies have been extensively studied, including organic materials, organic frameworks, noble metal nanostructures, carbon-based nanomaterials, and other nanomaterials.

Gold nanoparticles (AuNPs) can be utilized in PPT in forms such as gold nanospheres, nanocages, nanoshells, and nanorods due to their exceptional ductility. According to Mie theory, localized surface plasmon resonance (LSPR) can significantly enhance the absorption and scattering of electromagnetic radiation by noble metal NPs (27). The tunable LSPR properties of AuNPs significantly enhance light absorption, leading to a pronounced photothermal effect under irradiation. This unique feature enables the design of novel PTAs for cancer molecular imaging and PTT. Their photothermal conversion capability depends on particle size, shape, particle spacing, metal type, and local dielectric constant. For instance, gold nanostars exhibit the lowest cytotoxicity, the highest cellular uptake, and the highest heat production in MCF7 cells (28). Therefore, the optical properties can be adjusted by regulating the aforementioned factors to meet the requirements of practical applications. For instance, Chunyu Zhou et al. reported that silica-coated self-assembled gold nanochains (AuNCs@SiO2) exhibited excellent NIR-II deep tissue penetration photoacoustic imaging and photothermal therapeutic effects in tumor tissues, with a photothermal conversion efficiency as high as 82.2% (29). Additionally, in an experiment, Mendes et al. found that 14-nanometer spherical gold NPs exposed to visible green lasers could increase the toxicity of doxorubicin (DOX) to BC and selectively destroy cancer cells (30). This project demonstrated that the synergistic effect between the thermal effects generated by AuNPs and the cytotoxic action of DOX could reduce the survival rate of BC cells. The AuNCs@PDA-Mn NPs developed by Tingyu Yang et al. effectively eradicate tumor cells through photothermal conversion. Simultaneously, they accelerate the Fenton reaction process to enhance hydroxyl radical (·OH) generation for augmented tumor therapy (31). This study combined hyperthermia and ROS-mediated dual damage to tumor-associated antigens (TAAs), synergically enhancing innate and adaptive anti-tumor immunity.

Indocyanine green (ICG) is the only NIR organic dye approved by the U.S. Food and Drug Administration (FDA) for clinical medical imaging and diagnosis (32). Recently, many polymers and inorganic substance have been introduced to address the inherent issues of ICG degradation, rapid blood clearance, and *in vivo* imaging. For instance, the ICG and CTS-loaded HA-coated ZIF-8 drug delivery system ((ICG+CTS)@HA-ZIF-8) prepared by Zhe Li et al. exhibits excellent photothermal performance (33). This nano-preparation downregulates glycolysis-related proteins (PKM2 and HSPs), inhibiting tumor glycolysis and overcoming their heat resistance. Such modulation significantly enhances the efficacy of ICG-guided image-directed PTT in BC. However, Feng and his colleagues found that compared with ICG-paclitaxel (PTX) nanoparticles (ISPN), the expression of Calreticulin (CRT) induced by free ICG was not very significant and was 2.0 times lower than that of the ISPN+L group (34). This suggests that free ICG alone may have limited immunogenic effects, emphasizing the need for improved formulations. Juncheng Xuhong et al. developed a nanomedicine named “P/ICG” by integrating ICG and pyrotinib. This nanomedicine specifically targets HER2-positive BC cells, addressing the challenges of ICG in terms of targeting and light source dependency (35). The latest research has found that Liuhai Zheng et al. used a nanobody Nb215 with high affinity to bind to human and mouse Calreticulin (CALR). This approach targets the immune cell death (ICD) marker CALR. The combination of CALR nanotide-modified EcN-215 and ICG reshaps the tumor microenvironment (TME) by enhancing the infiltration of CD45+CD3+ T cells and CD11b+F4/80+ macrophages. This also means that the process by which CALR transfers from the endoplasmic reticulum (ER) to the cell surface after ICD induction can serve as a target for PTT in BC (36).

Carbon nanotubes are nanostructures with unique optical and electronic properties, as well as an ultra-high specific surface area. Due to these characteristics, they are considered versatile nanomaterials with applications in tissue engineering, thermal ablation, and drug delivery (37, 38). Carbon nanotubes are three-dimensional structures composed of graphene. Depending on the number of layers, graphene can be classified into single-walled carbon nanotubes (SWCNTs), double-walled carbon nanotubes (DWCNTs), and multi-walled carbon nanotubes (MWCNTs). Carbon nanotubes exhibit strong absorption capabilities in the near-infrared region, making them suitable for PTT in tumors. Particularly, SWCNTs have been successfully used for PTT or photoacoustic therapy targeting cancer cells by converting NIR radiation into thermal or acoustic energy (39–41). In an experimental study, a temperature-sensitive CNTs-PS/siRNA composite was synthesized using SWCNTs and MWCNTs modified by sucrose laurate and peptide lipids. This material has excellent photothermal conversion efficiency and anti-tumor activity, and is a potential nanocarrier for the combined application of gene therapy (GT) and PTT. The needle-like structure of carbon nanotubes facilitates the transmembrane penetration and intracellular accumulation of drugs through the “nano-edge” mechanism. Moreover, CNTs possess exceptional drug-loading capacity due to their high aspect ratio and surface area, making them an ideal platform for drug adhesion through covalent and non-covalent interactions. For instance, researchers have developed a drug delivery system based on MWNTs, which employs hydrophilic diamino triethylene glycol as a spacer between the nanotubes and the drug components (41). The CNTs are covalently conjugated with the antitumor drug 10-hydroxycamptothecin (HCPT), forming MWNT-HCPT conjugates that exhibit excellent antitumor activity in both *in vitro* and *in vivo* experiments (42). To enhance the antitumor efficacy of PTT, Li et al. utilized glycosylated chitosan (GC)-modified SWNTs for the treatment of metastatic BC in mouse models. In this study, the GC on the surface of the nanomaterial stimulates the host immune cells, enhancing the anti-CTLA inhibition (43).

PTA based on NPs are simple to prepare, exhibit strong chemical reactivity, and possess excellent biocompatibility, making them highly promising for applications in cancer therapy. Effective PTAs can penetrate deep into tumor tissues to eradicate cancer cells while causing minimal damage to healthy tissues. Adjusting the size, structure, and morphology of PTA to enhance the photothermal conversion efficiency is a crucial strategy for improving the efficacy of PTT. Image-guided PTT based on specialized PTAs can significantly enhance the accuracy of cancer diagnosis and treatment efficiency, enabling real-time monitoring and optimization of NIR irradiation conditions based on PTAs. Additionally, the integration of multiple modalities such as photothermal, IR-II fluorescence, photoacoustic, and magnetic resonance imaging represents the future trend in the diagnosis, treatment, and prognosis of BC.

## 3 The immune response of PTT on BC

**Figure 1:**
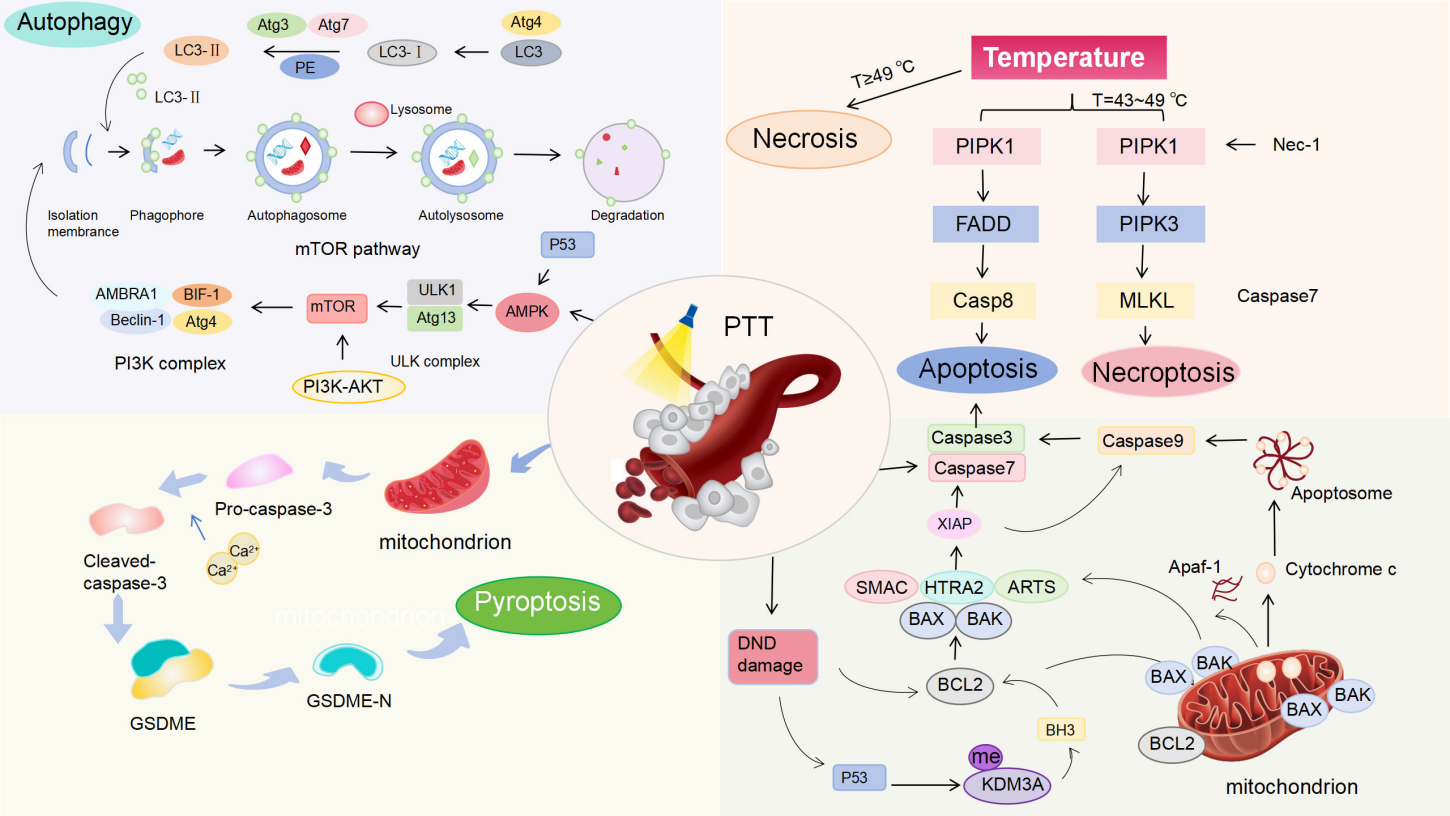
Mechanisms of PTT-induced cell death in BC. Under laser irradiation, PTT generates heat that induces ICD in a temperature-dependent manner. Necrosis dominates at the temperature of ≥49 °C. Apoptosis and necroptosis occur at the temperature of 43–49 °C. Molecular pathways: PTT causes DNA damage, activating p53 to modulate the Bcl-2/Bax pathway, triggering Cyt c release, caspase-3/9 activation, and apoptosis. PTT activates AMPK/p53, inhibiting the PI3K/AKT/mTOR pathway to induce autophagy. PTT-generated heat activates caspase-3, cleaving Gasdermin E (GSDME) to initiate pyroptosis.

The immune response of PTT to tumors refers to a series of physiological reactions such as PTT inducing tumor cell death to release antigens, antigen presentation, lymphocyte activation, immune molecule formation and the occurrence of immune effects (44). In fact, the immune effect is different from the immune response, referring to the direct execution of killing or clearance functions in the immune response. However, the specific mechanisms of cell death induced by PTT remains to be fully elucidated. Consequently, we analyzed the various pathways of cancer cell death induced by PTT to provide the exact foundation for understanding tumor immune antigens. These mechanisms enable us to explore improved strategies for enhancing PTT treatment of BC from multiple perspectives. Additionally, they provide theoretical support for the combination of PTT with other therapeutic approaches (**Figure 1**).

### 3.1 Antigen release

#### 3.1.1 Cell necrosis

When hyperthermia temperatures exceed 50 °C, tumor cells exhibit swelling, rupture of the cell membrane, and collapse of organelles, which inhibit tumor cell growth (45). However, the disruption of cell membrane function results in the release of excessive inflammatory mediators, such as interleukin IL-6, HSPs and nucleic acids. These released mediators can subsequently promote tumor growth (46–48). For instance, studies have shown that excessive inflammation can induce high expression of macrophage polarization factors. This subsequently leads to macrophage polarization to the M2 phenotype and facilitating new angiogenesis in inflammatory breast cancer (IBC) (49). Additionally, inflammation can enhance the infiltration of immunosuppressive cells and the inhibition of T cell responses (50). Furthermore, cell necrosis may contribute to cancer recurrence and metastasis (51). Cell death at this temperature cannot be regulated by life processes and is prone to cause significant damage to surrounding normal tissues. To address this issue, Gangqiang Yuan et al. developed a biosafe PTT therapeutic strategy, which precisely treats tumors through intratumoral self-assembly of renal-clearable AuNPs at the tumor site via host-guest interactions. Host AuNPs functionalized with both cyclo (Arg-Gly-Asp-D-Phe-Cys) and cyclodextrin interact with guest AuNPs simultaneously functionalized with both pH-responsive doxorubicin and adamantane, resulting in the self-assembly of renal-clearable AuNPs at the tumor site (52). This nanoformulation specifically targets the TME, avoiding damage to surrounding normal tissues.

An increasing number of studies have demonstrated that necrosis, apoptosis, and necroptosis occur simultaneously at temperatures ranging from 31°C to 39 °C. This thermal sensitivity has been further demonstrated in PTT, where GNR-FA induced PPT mainly induces tumor killing through necrosis and apoptosis at a high temperature of 46 °C (53). PTT triggers necroptosis via the death domain receptor (DR) signaling pathway at these temperatures, representing a PCD pathway independent of caspase activation. Necroptosis offers a novel strategy for reducing necrosis and enhancing tumor ablation in cancer phototherapy (54). Tumor necrosis factor-1α (TNF-1α) activates PARP1 through DNA damage, resulting in ATP depletion and subsequent necrosis (55). Notably, receptor-interacting protein 3 (RIP3) appears to be a pivotal protein in mediating TNF-α-induced cell necrosis. And RIP3 phosphorylates threonine 357 (T357) and serine 358 (S358) of mixed lineage kinase domain-like protein (MLKL), thereby facilitating cell necroptosis (56). With TNF-α and RIPK1 triggered by PTT, the expression of effector factors is significantly altered, demonstrating substantial cross-talk between the RCD pathways. For example, the expression of critical factors associated with necrosis signals (PARP1, RIPK1) is upregulated in cells, while autophagy-related genes induce autophagy-related cell death. Recent studies have shown that the therapeutic immune response caused by necroptosis can be driven by neoepitopes, including four mutated Major Histocompatibility Complex class II (MHC II) binding peptides and one MHC Class I (MHC I) binding peptide (57, 58). An interesting study reported that both apoptotic and necroptotic CT26 cells could effectively protect mice from the challenge of another AH1-expressing cancer cell line, 4T1 BC. The adaptive anti-AH1 immune response produced by CT26 cells stimulated by apoptosis was not as significant as that stimulated by necroptosis (59). The broader anti-tumor immune response of necroptosis to the new epitopes within the tumor has broken our previous understanding of necroptosis.

#### 3.1.2 Apoptosis

It has been demonstrated that PTT can induce another PCD pathway: apoptosis, when exposed to low temperatures (42-45°C) (60). This apoptotic pathway mitigates inflammation that may arise from the leakage of cytoplasmic contents due to compromised plasma membrane integrity, thereby reducing the adverse effects of PTT associated with elevated temperatures. Currently, two well-studied apoptotic pathways exist: exogenous pathways activated by death receptors and endogenous pathways initiated by mitochondrial signals. TNF receptor superfamily member 6 (Fas) and TNF-related apoptosis-inducing ligand (TRAIL) can rapidly trigger apoptosis upon binding with their respective ligands. These ligands are secreted by immune cells, particularly cytotoxic T lymphocytes (CTLs) (60). Target cells infected by pathogens can be induced to undergo apoptosis via this pathway, while damaged cells trigger their own apoptosis by Fasl/Fas.

Compared with exogenous pathways, mitochondria have been proven to play a key role in the regulation of endogenous apoptosis. Local hyperthermia induces endogenous stimuli such as oxidative stress, DNA damage, and the unfolded proteins accumulated in the endoplasmic reticulum (ER) (61–63). Stimulated mitochondria release cytochrome c (Cyt c) through their outer membrane into the cytoplasmic matrix. The released Cyt c then binds to apoptotic protease activating factor-1 (Apaf-1), initiating the cascade of endogenous cell apoptosis. Additionally, mitochondrial gap proteins have been found to be released into the cytoplasmic matrix, relying on the increased permeability of the mitochondrial outer membrane. Among these factors, the permeability of the mitochondrial outer membrane is crucial for internal apoptosis in tumor cells. This permeability is primarily regulated by the interactions of B-cell lymphoma-2 (Bcl-2) family through their polymerization and depolymerization (64). However, cells containing Bax/Bak are unable to undergo apoptosis during serum deprivation, loss of attachment, and growth factor withdrawal, indicating that growth factors are essential for mediating Bax/Bak protein to initiate the mitochondria-dependent apoptosis pathway (65). The occurrence of tumor cells has been closely linked to the Bcl-2 family. The p53 gene regulates the upstream pathways governing the expression of the Bcl-2/Bax proteins, and inhibits the downstream pathways of Bcl-2 while activating Bax protein through the BH3 signaling pathway. Through these dual regulatory mechanisms, p53 effectively prevents cell apoptosis (66). Tumor cells can evade apoptosis through the deletion and mutation of the p53 gene. Therefore, reactivating p53 gene expression and targeting its downstream pathways are considered potential strategies for inducing apoptosis. For example, Yingfang Xing et al. found that plasma-NP-mediated photothermal therapy (PPTT) operates through the same apoptosis mechanism as H2O2. This mechanism specifically involves activation of the nucleo-coding protein Bak and inhibiting the Bcl-2 protein (64). After 15 minutes of irradiation, the proportion of dead cells following PPTT increased significantly, reaching 94.8% of the total, while the expression of caspase 3 rose threefold. Cyt c triggers the caspase cascade reaction via apoptotic bodies, ultimately leading to the apoptosis of tumor cells. In general studies, the regulation of caspase-3 and caspase-9 activity is employed to induce cell apoptosis in BC. In addition to heat resistance, Hsp27 regulates apoptosis by interacting with Akt to inhibit caspase-3 and caspase-9 activity, thereby contributing to tumor cell survival (67). The pro-apoptotic effect of the Hsp90 inhibitor NVP-AUY922 has been utilized to enhance the down-regulation of Hsp72 or Hsp73 and to regulate the activity of caspases 3/9, inducing cell apoptosis in BC (68). Furthermore, the study found that the epigenetic function of BC affects the sensitivity of photothermal efficacy to some extent. Lysine methyltransferase (KMTs) and lysine demethylase (KDMs) interact in the efficacy of photothermal ablation of BC by balancing the methylation of histones. KDM3A indirectly inhibits the transcription of pro-apoptotic genes by removing the methyl group from p53-K372me1, thereby alleviating its pro-apoptotic function (69). Consequently, it is considered effective for the photothermal efficacy of BC to downregulate KDM3A and reduce p53 occupancy at PUMA and NOXA loci, inhibiting their expression (69).

Recent findings indicate that exogenous pathways activated by death receptors are also interrelated with endogenous pathways initiated by mitochondria. In HR-positive patients, tumor necrosis factor TNF-α binds to its corresponding receptor, leading to an increase in the expression of p53 and Cip1 (tumor suppressor proteins and cell cycle inhibitors). This increased expression consequently obstructs the G1 phase of the cell cycle. Concurrently, TNF-α facilitates the release of Cyt c from mitochondria of ER-positive cancer cells, triggering apoptosis (70). One study demonstrated that heat stimuli can simultaneously inhibit the phosphorylation of STING and IRF3 proteins in 4T1 cells, resulting in the down-regulation of the STING protein and inducing apoptosis in BC (71). During hyperthermia, the activation of the STING signaling pathway is suppressed, which may represent a self-regulatory mechanism in 4T1 cells to mitigate apoptosis induced by hyperthermia (71). Consequently, combining PTT with STING agonists may enhance the efficacy of heat-induced apoptosis and bolster anti-tumor immunity.

#### 3.1.3 Autophagy

Autophagy serves as a mechanism to mitigate cell death by eliminating damaged organelles that may trigger apoptosis, thereby preventing the accumulation of harmful metabolites during periods of stress (72). However, this compensation mechanism over-activated can result in cellular destruction and complete organelle elimination. Therefore, autophagy can either inhibit or promote the tumor development, indicating an environment-dependent role for autophagy in cancer. Autophagy can be categorized into microautophagy, macroautophagy and chaperone-mediated autophagy (CMA), with CMA and macroautophagy primarily occurring in PTT. Macroautophagy is characterized by the encapsulation of entire cellular regions within a double-membrane vesicle known as an autophagosome, which subsequently fuses with the lysosome to form an autophagolysosome. Light chain 3 (LC3) and the increased ratio of LC3-LL/LC3-L are recognized as biomarkers of autophagy. Additionally, p62 is a protein that is specifically degraded through autophagy, serving as a reflection of autophagic flux. Defects in autophag are associated with genomic damage, metabolic stress, and an increased susceptibility to tumorigenesis (73). Furthermore, Julian J and coworkers found that macroautophagy in Bax/Bak cells is induced by growth factor withdrawal, breaking down intracellular substrates to promote cell survival (74). A substantial body of evidence indicates that growth factor deprivation is closely linked to viral infections, ER stress, toxins, and chemotherapeutic-induced autophagy (75–77). At present, the autophagy pathways in tumor cells are relatively complex, with the AMPK/mTOR/p70S6K signaling pathway and the Beclin-1 signaling pathway being particularly relevant to autophagy in BC (78). Beclin-1 induces autophagy by binding to an evolutionarily conserved domain and activating vacuolar protein sorting 34 (Vps34). It has been observed that the inhibition of Beclin-1 phosphorylation affects autophagy in HER2-positive cancer cells (79, 80). Compared to other subtypes of BC, the lipidation of Beclin1 and the microtubule-associated proteins light (LC3A and LC3B), which are highly expressed in triple-negative breast cancer (TNBC) cells, demonstrates significant inhibition of cell growth (81). This finding provides a promising direction for studying autophagy in TNBC tumor cells. Besides, autophagy promotes the regulation of tumor suppression by Beclin-1 through its interaction with UVRAG and Bax interaction factor-1 (Bif-1). This interaction enhances the binding between Beclin-1 and Vps34, resulting in increased autophagy (82). Notably, single deletions or mutations of UVRAG, along with reduced expression of Bif-1 have been observed in BC, potentially impairing autophagy-mediated tumor suppression (83). This study shows that the binding of Bif-1 to UVRAG-Beclin-1 complex has significant potential as a potential activator of phagocytosis and tumor suppressor.

Autophagy is rapidly upregulated to produce intracellular nutrients or to reshape cellular structure s through the activation of AMPK and p53 signaling pathways. Conversely, growth factors can inhibit autophagy by acting on the PI3K (phosphoinositide 3-kinase)/AKT (protein kinase B)/mTOR (mechanistic target of rapamycin) pathway. mTOR is a critical molecule in the regulation of autophagy. AKT phosphorylated by the PI3K complex tuberous sclerosis 1/2 (TSC1/2), initiates the AMPK pathway as a tumor suppressor by blocking mTOR activation (84, 85). Shengliang Li et al. employed Ctab-based Au NRs in PTT to induce the rapid downregulation of p62 through starvation. This downregulation subsequently activated the AMPK/mTOR/p70S6K signaling pathway to promote autophagy in BC (84). Thus, the photothermal effect enhances the accumulation of autophagosomes by activating autophagic flux rather than blocking their formation. Furthermore, it has been observed that autophagy in tumor cells is closely associated with elevated concentration of reactive oxygen species (ROS). These elevated ROS concentrations promote autophagy by inducing DNA damage, mitochondrial membrane degradation and ER stress. Shin-Hyung Park and his colleagues further found that ROS promotes the production of autophagy through PI3K/Akt signaling pathway (86). However, autophagy serves to protect against the degradation of tumor cells and the recovery of damaged organelles in response to cellular damage. Additionally, autophagy also supports the recycling of nutrients, which collectively diminishing the therapeutic efficacy of PTT on cancer cells (87). As a result, autophagy is widely recognized as a mechanism that enhances tumor cell survival by improving stress tolerance, which in turn provides cancer cells with essential nutrient and energy requirements. In addition, hypoxia induces autophagy in a HIF-1α-dependent manner, contributing to tumor survival (88). At present, inhibition of autophagy through genetic or pharmacological methods has been widely shown to increases the sensitivity of tumor cells to PTT. For instance, the deletion of the autophagy gene FIP200 has been found to reduce the growth of breast tumors (89). A large number of studies have found that autophagy inhibitors target LC3, p62, beclin-1 and autophagy associated gene 5 (Atg5) in conjunction with PTT, while inhibiting the AKT/mTOR signaling pathway, significantly enhancing the autophagy activity in tumor cells (90, 91). The reduction of ATG-related genes enhances the responsiveness of cancer cells to various autophagy inhibitors, utilized in cancer treatment (92). The enzymes ATG7 and ATG3 activate ATG4. ATG3 functions as part of a protein complex that includes ATG5, ATG12, and ATG16. LC3 subsequently covalently binds to phosphatidylethanolamine. It associates with the autophagosome surface until ATG4 partially removes and recycles it (93). With autophagy inhibition overcoming the heat resistance of cancer cells, Shao et al. designed a multifunctional ratcheted structure NP incorporating the autophagy inhibitor chloroquine (CQ) alongside the glucose consumer glucose oxidase (GOx) (94). The NP (PDA@hm@CQ@GOx) synergistically combined low-temperature PTT (≤ 45°C) with energy metabolism regulation and autophagy inhibition, significantly enhancing the expression and accumulation of LC3 II in the cytoplasm. Xiaoqing Ren et al. found that the number of autophagosomes in MCF-7 was proportional to the laser illumination time, because CQ inhibited the degradation of autophagosomes (95). Despite the significant anti-tumor potential of PTT, recurrence due to incomplete tumor ablation presents a significant challenge in tumor treatment. The study found that the fusion of autophagosomes with lysosomes degrades antigens and diminishes the anti-tumor immune response during the formation of autophagosomes, suggesting that the ability of tumor cells to evade the immune system is closely linked to the process of autophagy (96). End-autophagy inhibitors are typically used to improve antigen presentation by up-regulating MHC-I in tumor cells (97). Therefore, the upregulation of MHC-I to enhance DCs antigen presentation is regarded as a crucial strategy to mitigate the weakening of immune response induced by PTT-mediated autophagy.

Recently, the CMA signaling pathway as an additional mechanism by which PTT influences tumor autophagy. In this pathway, cytoplasmic proteins featuring a pentapeptide motif (KFERQ-like motif) are recognized by the chaperone 71 kDa protein (HSPA8), which facilitates their degradation via lysosomes (98). HSPA8 forms a partner/substrate complex with HSP90AA1, a cytoplasmic class A member 1 co-partner. Research by Maryam Ghafarkhani revealed the expression of HSPA8 was significantly upregulated (5.45 times) at 48°C, indicating that CMA was closely associated with the temperature of PTT (99). Consequently, controlling the temperature of PTT may represent a promising strategy for BC treatment by integrating CMA and macroautophagy. Furthermore, the chaperone system (CS), the ubiquitin-proteasome system (UPS) and CMA collectively influence the autophagic processes in tumors, playing crucial roles in maintaining protein homeostasis (67).

#### 3.1.4 Pyroptosis

Pyroptosis is a highly inflammatory form of PCD that activates the inflammatory response and restructures TME. This process can be categorized into typical and atypical pathways. In the typical pathway, inflammatosomes are triggered by intracellular pathogens and DAMPs, leading to release of inflammatory cytokines and chemokines. This promotes the assembly of the NLRP3 inflammasome and affects macrophages polarization (100, 101). Regulating the activity of caspase-1 can also modulate macrophage differentiation, with low activity inhibiting the differentiation of monocytes into macrophages (102). Conversely, the atypical pyroptosis pathway does not involve inflammasome. At present, phototherapy has been shown to induce atypical pyroptosis, thereby enhancing tumor immunotherapy. The heat generated at the tumor site disrupts cellular homeostasis, triggering caspase-3 activation. Caspase-3 then cleaves GSDME ultimately inducing pyroptosis (103, 104). GSDME, a critical protein in pyroptosis, typically exists in a self-inhibited state and induces cell pyroptosis through the formation of membrane pores in the gas protein-N domain (105). The formation of GSDMD pores in the membrane disrupts osmotic potential, leading to cell swelling and eventual lysis. Additionally, MLKL is also thought to play a direct role in membrane lysis during pyroptosis (106). The pro-inflammatory contents released by pyroptosis contribute to systemic inflammation and provide favorable conditions for solid tumor immunotherapy (105, 107). HMGB1 and CRT are released from the nucleus into the cytoplasm, accompanied by significant morphological changes, highlighting the anti-tumor immunity mediated by pyroptosis (104). Inflammatory pyroptosis promotes DCs maturation, enhancing tumor antigen presentation. One study suggested that PTT also influences the proliferation, migration and survival of BC cells by the transient the transient receptor potential vanilloid 1 (TRPV1) channel. The photothermal facilitates the gradual infiltration of Ca^2+^ into tumor cells, activating caspase-3 and promoting the release of Cyt c, thereby inducing pyroptosis (104). This Ca^2+^-dependent pyroptotic cascade was quantitatively validated in some comparative studies. For example, Due to the infiltration of extracellular Ca^2+^, BNP + photoactivation induced a 25.8-fold increase in cytoplasm, which was 3.5 times, 6.0 times, 1.6 times, and 1.2 times higher than naked ICG, DCT, ICG+DCT, and IDNP (103). Compared with the negative control, the intracellular caspase-3 activity increased by 1.6 times, 2.0 times, 4.4 times, 5.7 times, and 4.7 times, respectively. Ca^2+^ mainly enters tumor cells through membrane puncture induced by photoactivation. Currently, the application of chemotherapeutic drugs that induce pyroptosis is limited in the biomedical field due to issues of resistance and significant side effects (108). Inflammatory pyroptosis promotes DCs maturation and enhances the infiltration of CD8+ T cells into tumors, thereby overcoming immune evasion through pro-inflammatory factors. This provides a theoretical foundation for the combination of PTT with chemotherapy. For example, research on TNBC lung metastasis demonstrates that PolyMN-TO-8 under laser exposure induces moderate COX-2 elevation and disrupts immune escape mechanisms. This effect promotes the infiltration of CD8+ T cells both into tumors and lungs, further preventing the formation of tumor nodules (109). Similar amplification of therapeutic effects occurs through protein modulation in other systems. Notably, the severe thermal denaturation of dihydrofolate reductase (DHFR) induced by Methotrexate amplifies the inhibitory effects of Methotrexate on DHFR.

#### 3.1.5 Limitations of HSPs

Although PTT exhibits good spatiotemporal controllability, its effectiveness is limited by heat resistance in BC. Heat tolerance serves as a protective mechanism mediated by HSPs, which are significantly upregulated in response to adverse external stimuli such as hyperthermia or toxic chemicals. This upregulation corrects protein misfolding and maintains protein activity. HSP70 and HSP90, critical proteins involved in cellular heat tolerance, are overexpressed in many tumors (110, 111). The survival of tumor cells is highly dependent on the intrinsic self-defense pathways mediated by HSPs in BC. Studies have demonstrated that inhibiting HSP function disrupts cell homeostasis and interferes with the integrity of protein interactions, thereby reducing heat resistance (6). However, the precise mechanisms for dismantling these self-defense pathways to enhance photothermal efficacy are still under investigation. Research has indicated that Hsp27, Hsp60, Hsp70, and Hsp90 are promoters of BC tumorigenesis and are closely related to tumor aggressiveness, EMT, metastasis, and treatment resistance (5, 6). Further studies have revealed that Hsp70 plays an anti-apoptotic role by inhibiting the activation of caspase-3, thereby blocking the stress-activated kinase pathway (112). PTT treatment induces increased expression of mitochondrial HSP70 (mHsp70) on MCF-7 cell surfaces compared to normal conditions. The upregulated mHsp70 subsequently aggregates or gets released into the extracellular environment in certain areas of the cell membrane (113). Meanwhile, HSP70 and HSP90 are involved in the processing of intracellular antigens and the presentation of membrane-anchored antigens to stimulate immune cells. In fact, a study found that the ability of PTT to induce DNA double-strand breaks may be a result of the changes in heat shock protein expression caused by PTT (114). In conclusion, localized heating enhances the expression of heat shock protein in BC cells, leading to DNA damage and increased heat resistance. Consequently, investigating strategies to disrupt self-defense mechanisms and promote tumor cell death is a crucial step in the advancement of PTT.

#### 3.1.6 The connections among different ways of cell death

The thermal effect of PTT leads to different ways of cell death through multiple molecular pathways, which may dominate under different conditions. For example, the influence of temperature. Mild hyperthermia (43-49 °C) may induce necroptosis and apoptosis more, while high temperature (≥49°C) leads to necrosis (54, 60). During the treatment process, autophagy has the opposite function of inhibiting tumors or promoting tumors. Under brief extreme high temperatures (42-45 °C, 5–20 min), the induced GFP-ATG8 punctate structure was co-located with the Golgi apparatus (115). Excessive activation of autophagy may trigger autophagy. However, in actual research, it is mostly PTT systems at mild temperatures. No correlation has been found between the tumor-promoting effect of autophagy and the temperature of PTT for the time being. Only correlations with tumor type, tumor stage, tumor microenvironment and composition of nanomaterials have been found (116). Meanwhile, the thermal effect may simultaneously trigger multiple death pathways, and there may be cross-communication among different pathways. For example, ROS can not only cause mitochondrial damage leading to apoptosis, but also activate NLRP3 leading to pyroptosis. Additionally, ROS promote the generation of autophagy in cells through PI3K/Akt signaling pathway (60, 86, 117). Autophagy also regulates tumor suppression through the interaction between Beclin-1 and Bcl-2. At the same time, autophagy has cross-regulation with the outer mitochondrial membrane pathway that regulates apoptosis (65). Furthermore, both apoptosis and pyroptosis rely on Caspase-3 mediating cell death. Meanwhile, GSDM serves as a switch molecule in apoptosis and pyroptosis transformation (118). When GSDM is highly expressed, it can induce tumor cell death through caspase-3-dependent pyroptosis. When the expression is low, the cell death pattern changes to apoptosis (119, 120). Furthermore, when exogenous apoptosis cannot be initiated due to genetic, molecular or pharmacological interference, the pro-death signals originally intended to trigger apoptosis now cause necrotizing apoptosis (121, 122).

### 3.2 Activation of immune cells

**Figure 2:**
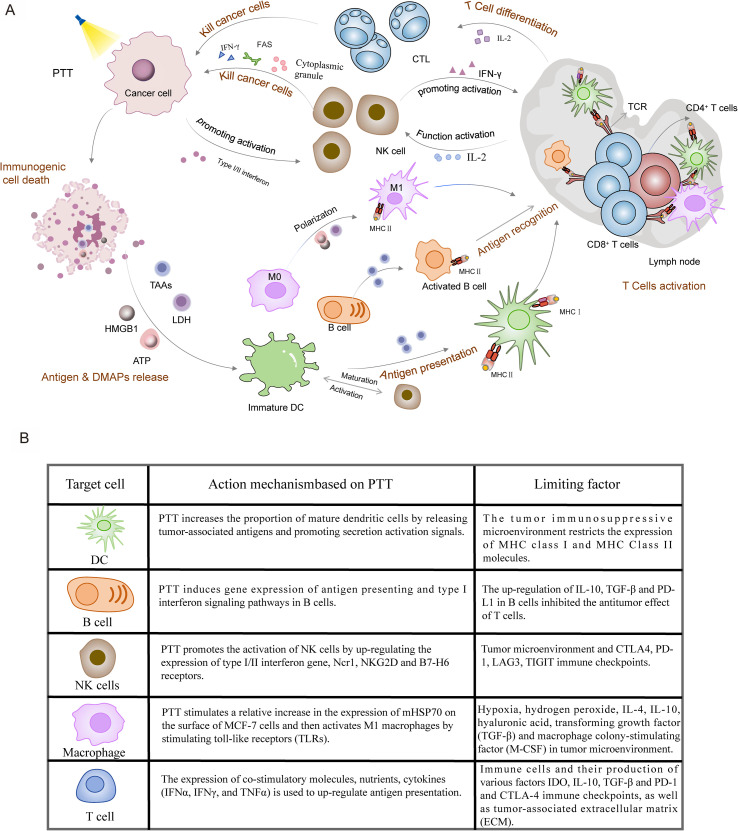
PTT-mediated activation of anti-tumor immune response. **(A)** PTT generates localized heat under laser irradiation, inducing ICD in tumor cells. ICD leads to release of DAMPs (ATP, HMGB1, LDH) and TAAs, which are captured by APCs including DCs, macrophages, and B cells. Activated APCs prime T cells, promoting their differentiation into tumor-lytic T cells (TLCs). Concurrently, T cell-derived interleukin-2 (IL-2) enhances the cytotoxic activity of NK cells, establishing a comprehensive anti-tumor immune response. **(B)** The mechanisms of action of immune cells, including DCs, B cells, NK cells, macrophages, and T cells, in the role of PTT in BC, as well as the limitations they face.

PTT-mediated immunotherapy is a promising approach to cancer treatment that activates both the innate and adaptive immune system. The generation of an anti-tumor immune response involves the following critical steps: Photothermal stimulation, oxidative stress, DNA damage and high levels of ROS inducing the aforementioned ICD, which releases DAMPs and TAAs. This process further activates both the innate and adaptive immune systems (24). DAMPs include HMGB1, CRT, HSP90, HSP70, and ATP (123). Following heat damage, HMGB1 and ATP are released as ‘find me’ signals to recruit APCs. Meanwhile, CRT exposure on the cell membrane surface acts as an ‘eat me’ signal to promote phagocytosis by APCs (124). In addition, APCs can be effectively activated by HSP antigen complex formed by HSP binding to TAAs. DAMP is recognized by pattern recognition receptors (PRRs) on the surfaces of macrophages, DCs, and B cells (125). DCs subsequently migrates to draining lymph nodes where they present tumor antigens and stimulate naive T cells. The tumor antigens within MHC-I and MHC-II molecules are cross-presented to CD8+ T cells and CD4+ T cells, initiating the adaptive immune response (126). Meanwhile, TAAs are taken up and presented by DCs, which can also activate T cell-dependent adaptive immunity. NK cells initiate innate immune phagocytosis or tumor cell killing through cytotoxicity or the secretion of IFN-γ (127, 128). Therefore, ICD can significantly enhance the tumor immune response. Importantly, ICDs effectively serve as a bridge between PTT and immunotherapy for cancer treatment. However, little is currently known about how PTT specifically affects immune cells. Therefore, we analyzed the mechanism by which PTT-induced cell death activates the immune response, revealing the complexity of this process (**Figure 2A**).

#### 3.2.1 Cellular immunity of T cells

##### 3.2.1.1 Antigen presentation

DCs play a crucial role in in initiating, regulating, and maintaining the immune response within the human immune system. There are two main types of DCs, conventional dendritic cells (cDCs) and plasmacytoid dendritic cells (pDCs). As traditional mature DCs, cDCs expresses high levels of MHC-II molecules, co-stimulatory molecules and other signaling factors to activate naïve T cells. It has been demonstrated that pDCs recognize pathogen-associated molecular patterns (PAMPs) through receptors such as Toll-like receptor 7 (TLR-7) and Toll-like receptor 9 (TLR-9), leading to the release of significant amounts of interferon (129). cDCs serve as highly specialized APCs, and their maturation stage is critical for T cell activation (130). PTT enhances the proportion of mature DCs by releasing TAAs and promoting activation signals (**Figure 2B**). The TAAs present in tumor fragments post-PTT can function as an *in situ* cancer vaccine, which are captured by DCs and presented on MHC- II molecules (131). However, tumor thermal ablation often fails to generate sufficient antigen, thereby limiting the complete maturation of DCs. Therefore, increasing the availability of TAAs through more frequent applications of PTT in tumors has the potential to strengthen the vaccine-like functions (132). Yet, antigen availability alone may be insufficient to achieve full DCs maturation, as the activation threshold also depends on co-stimulatory signals.

Precisely this dual requirement is addressed by exogenous signaling molecules. Beyond TAAs, DCs can be activated by lipopolysaccharides, TLR ligands, antibodies (Abs), and, pro-inflammatory cytokines, with synergistically enhance vaccine-like functions (133, 134). The cytokines released by mature DCs, including TNF-α, IL-6 and IL-12, rare closely associated with the activation of T cells (135). Mechanistically, DCs interact with T cells through co-stimulatory molecules, adhesion molecules, MHC, cytokines and chemokines. Of particular importance, the expression of MHC-II molecules is regulated by cytokines which modulate immune function and inhibit tumor growth. DCs are activated via P2 receptors and subsequently enhanced by inflammatory signals such as LPS, TNF-α, IL-1β, or IL-6, leading to the upregulation of co-stimulatory and adhesion molecules. Various cytokines, including TNF-α and IL-12, further promote the presentation of antigens by MHC molecules, thereby activating T cells, and inducing antigen-specific immune responses (134). While PTT targeting P2 receptors has not yet been applied in BC, it may represent a promising direction for improving PTT. In addition to intrinsic cytokines, novel nanomedical materials offer new avenues for enhancing immunotherapy responses related to antigen presentation (136). Recent studies have reported that NPs modified with nano spikes induce changes in potassium (K^+^) levels under mechanical stress. This ionic imbalance activates DCs inflammasomes via TLR-4 pathway, thereby promoting the maturation of DCs in TNBC (137). Additionally, these spike-modified NPs significantly enhance T cell and humoral immune responses. Inspired by these findings, Yanlin Feng prepared rod-shaped plasma Au-Pd heterostructures (Au-Pd HSs), integrating nanoscale spike structures and phototherapy. This innovative design enhances the photoimmune response through DCs maturation (137). Furthermore, the surface chemistry of NPs can influence the capture of TAAs. Yuanzeng Min discovered that AC-NPs leverage NP surface specificity to enhance protein antigen recognition. This property increases the presentation of tumor-derived peptide antigens (TDPAs) by APCs, ultimately activating CD8+ T cells (138). Notably, NPs modified with amine groups can bind to proteins through ionic interactions, forming antigen-trapping NPs (ACNs) that enhance the presentation of TDPAs by APCs. Besides, it has been reported that dextran derivative-based pH-sensitive liposomes can be delivered to the cytoplasm of DCs, triggering antigen-specific cellular immunity via the MHC-I pathway (139). However, their immunogenic potential ultimately depends on downstream DCs maturation, a process requiring both antigenic signals and co-stimulatory activation.

Studies have shown that the expression of IFN-g secreted by DCs and the presence of MHC-II/CD80 on the cell surface are enhanced by PTT, which promotes DCs maturation (140). Imiquimote (R837) and other immune adjuvants, such as glycated chitosan (GC), also assists PTT by enhancing antigen uptake by DCs and facilitating their delivery to T cells. This adjuvant effect amplifies tumor-specific T cell response to target residual tumor cells at the treated site (140). However, IFNs has been found to produce negative feedback effects, leading to T cell depletion and immune suppression (141). The regulation of IFNs to enhance DCs maturation and promote a positive immune response remains unclear.

In addition to exogenous signaling molecules, endogenous injury-related molecular patterns, such as HMGB1, CRT and HSPs with immunogenic characteristics, are closely associated with the maturation of DCs (142, 143). Among these, the interaction between HMGB1 and TLR4 is critical for DCs maturation (144). For instance, endogenous adjuvants, including ATP, CRT, and HMGB1, released by CpG-coated CpG PBNP promote the release of tumor antigens through PTT (145). However, their therapeutic potential in clinical settings is often limited by the immunosuppressive TME.

This challenge is particularly evident in TNBC, where despite advances in clinical trials, inadequate immune activation and autoimmune toxicity restrict patient responses (146). The tumor immunosuppressive microenvironment and other factors inhibit or down-regulate co-stimulatory molecules on the surface of DCs, resulting in the impaired expression of MHC-I/MHC-II molecules (147). Therefore, enhancing immune stress, both internal and external, can be achieved by improving the antigen uptake, processing, and presentation capabilities of DCs, as well as by remodeling the TME in BC treatment through PTT. The Antigen-presenting function of DCs primarily relies on MHC-I and MHC-II molecules which present TAAs from tumor fragments to activate T cells. Crarri et al. reported that 10% to 30% of tumor patients exhibited the deletion of MHC-I molecules on the surface of cancer cells, or defects in the expression of either the MHC-II heavy chain or light chain (148). Consequently, there is potential to enhance the antigen-presenting function by increasing the gene expression of MHC-II molecules in DCs. For example, RuYan Li developed PDA-IMQ-IND-PEI NPs based on PTT and the IMQ immune checkpoint blockade (ICB) system, which significantly upregulated the expression of MHC-II (149). The up-regulation of antigen presentation mediated by MHC-II molecules provides a theoretical foundation for PTT combining PTT with immune checkpoint therapy. However, reversing the inhibition of the tumor-suppressing microenvironment is contingent upon the successful activation of the immune system. Research has shown that an increase in the proportion of regulatory Tregs is negatively correlated with tumor progression, prognosis, and patient survival rates in the peripheral blood and local TME of various solid tumors, including BC, ovarian cancer, lung cancer, and liver cancer (150). Given that Tregs suppress anti-tumor immunity, strategies to remodel the immunosuppressive TME are critical. In this context, Ruijie Chen proposed that the iron-based NPs (nFeAPG) release APG and Fe^3+^, which can induce iron sag, generate ROS, and reduce the expression of programmed cell death ligand 1 (PD-L1). These coordinated actions collectively remodel TME to enhance anti-tumor immunity. On this basis, PTT significantly enhances the recruitment of DCs and alters the proportions of T cells, demonstrating notable anti-tumor immunotherapeutic effects (151). However, PTT may down-regulate antigen presentation under certain conditions, impacting tumor immunity. When PTT-mediated autophagy activation occurs in tumor cells, autophagosomes serve as effective vectors for tumor antigen cross-presentation. Lysosomal degradation of autophagosomes containing TAAs diminishes antigen presentation (152, 153). Therefore, the application of autophagy inhibition strategies, such as autophagy inhibitors, mitigate the degradation of TAAs and MHC-I molecules. This preservation of antigens enhances the maturation of DCs and potentially improves antigen presentation (96). A robust antitumor immune response requires coordinated activation of multiple immune cell populations, particularly B cells, which complement DCs functions through distinct mechanisms.

Indeed, beyond their classical role in antibody production, B cells have been shown to enhance the immune response by increasing the expression of genes associated with antigen presentation. B cells differentiate into plasma cells and APCs in response to antigens, performing functions such as antigen presentation, immunoglobulin secretion, and the direct killing of tumor cells. Furthermore, B cells produce cytokines that regulate T cells to suppress anti-tumor immune responses (154). PTT induces the expression of genes related to antigen presentation and type I interferon signaling pathways in B cells, resulting in an increased the proportion of TIBs (**Figure 2B**). Tumor-infiltrating CD20+ B cells exhibit TAAs, such as APCs, which can enhance the tumor-killing effect of T cells and significantly increase the secretion of immunoglobulin G (IgG) following PTT.

##### 3.2.1.2 Activation of T cells

At present, PTT research focusing on immune responses primarily emphasizes T cells in BC (155). T cells, which originate from bone marrow stem cells and differentiate in the thymus, are primarily responsible for mediating cellular immunity. CD8+ and CD4+ T cells play significant roles in orchestrating the immune response against tumors and are positively correlated with the survival rates of BC patients (156). Initial T cell activation and proliferation are facilitated by DCs through antigenic stimuli and co-stimulatory molecules. The T cell antigen recognition receptor (TCR) specifically binds to MHC molecules on the surface of APCs, achieving functional activation of immune lymphocytes in response to antigenic stimuli. In addition, co-stimulatory molecules are essential for enhancing the interaction between T cells and APCs during antigen recognition. Numerous studies have indicated that T cells can be adversely affected by the blockade or masking of antigens on the surface of tumor cells. Notably, research has demonstrated that PTT enhances the antigen recognition capabilities of T cells towards DCs by continuously promoting the production of the co-stimulatory molecule Icos (156). Among the activated T cells, a significant proportion consists of naive CD8+ T cells and central memory CD4+ T cells, indicating that photothermal tumor ablation fosters a robust anti-tumor immune memory. Additionally, the signaling molecules involved in T cell activation via PTT encompass not only co-stimulatory molecules but are also closely linked with cytokine expression in CD8+ and CD4+ T cells (156). Recent studies have identified nutrients as a fourth signaling molecule that regulates T cell activation. Quiescent T cells primarily require lower ATP levels, whereas effector T cells necessitate a high metabolic flux through growth-promoting pathways (157). Therefore, nutrients play a crucial role in the activation and increase of T cells, elucidating why PTT activates the AMPK/mTOR signaling pathway from the perspective of antigen presentation (158). However, the functional outcome of these cells is closely related to the TME, where immune-suppressive mechanisms override metabolic advantages, leading to T cell exhaustion.

Indeed, immune cells and extracellular matrix (ECM) components recruited and activated by tumor cells form an inflammatory suppressive microenvironment in the early stages of tumor colonization. However, the relevant effector cells often exist in a state of exhaustion or undergo remodeling within this microenvironment due to sustained antigen stimulation and immune responses. This progressive dysfunction ultimately fosters the development of an immunosuppressive environment (159). PTT enhances the pro-inflammatory signaling pathways in T cells, including IFN-α, IFN-γ, and TNF-α, thereby increasing the expression of tumor cell surface antigens. This process serves as a crucial mechanism for transforming ‘cold’ tumors into ‘hot’ tumors, promoting tumor inflammation through PTT and driving immune remodeling of T cells. Nevertheless, the T cell activation induced by PTT alone is constrained by pro-inflammatory cytokines. Therefore, it is imperative to explore new strategies that boost the secretion of pro-inflammatory factors to enhance anti-tumor immunity in conjunction with PTT. Guo et al. utilized hollow copper sulfide to bind with CpG oligodeoxynucleotides, significantly inducing the secretion of IFN-γ and IL-2, while also increasing the infiltration and drainage of NK cells and DCs (160). Moreover, the combination of PTT and immunoadjuvant can induce vaccine-like functions by downregulating Myc targets and G2M checkpoints (131). However, the interaction between tumor cells and immune cells within the inflammatory microenvironment, along with various immune checkpoints such as IDO, IL-10, TGF-β, PD-1, and CTLA-4, results in T cell dysfunction (161). Most studies indicate that PD-1/PD-L1 on T cells, CTLA-4 on Tregs, and CD47/signal regulatory protein-α (SIRPa) impede anti-tumor immunity (162). Additionally, the inhibition of PD-1/PD-L1 has been shown to enhance exposure to CRT, as well as the release of ATP and HMGB1 (163). Consequently, an effective strategy for PTT is to indirectly enhance the cytotoxic effect of T cells on both *in situ* and metastatic tumors by blocking these negative signals. For instance, Yijian Gao demonstrated that the combination of donor-acceptor conjugated diradical polymers TTB-2, PD-1 antibody, and PTT can effectively prevent lung metastasis of BC (164). Furthermore, some studies have indicated that PTT combined with anti-CTLA-4, utilizing the organic nanocomposite material PLGA-ICG-R837 can promote the transformation of memory T cells. This immunomodulatory effect effectively inhibits tumor recurrence and achieves long-term tumor-free survival (131). In certain cases, PTT may to some extent suppress anti-tumor immunity. Wang et al. found that PTT may increase the population of CD4+ T cells, a majority of which are regulatory T cells (Tregs), yielding results contrary to expectations (165).

In fact, BC cells and cancer-associated fibroblasts (CAFs) modify the TME, while promoting the growth of host blood vessels, thus altering the characteristics of ECM (161). Tumor-infiltrating lymphocytes (TILs) are typically obstructed by the interstitial regions within the TME rather than being distributed around tumor cells. This spatial restriction hinders their ability to induce cytotoxic T cell responses (136). Consequently, it is clinically significant to increase the permeability of therapeutic drugs and the proportion of TILs in deeper tumor regions by employing effective treatment methods to reshape the TME. Inspired by this, Yanan Tan utilized IR780/DPPC/BMS to disrupt the ECM barrier through hyperthermia, facilitating deeper penetration into the tumor (136). The reduction of CAFs surrounding tumor cells has been shown to enhance the distribution of CD8+ T cells (136). Interestingly, cationic IR780/DPPC/BMS can capture released TAAs, thereby improving the antigen presentation by DCs.

#### 3.2.2 Humoral immunity of B cells

Reports show that approximately 20% of BC cases exhibit elevated levels of B cells, with B-cell infiltration in malignant tumors, such as invasive BC or ductal carcinoma *in situ*, being significantly greater than in benign breast lesions (166, 167). Antigens, high endothelial venules and chemokines like CXCL13 are secreted to facilitate infiltration into the TME by BC cells. B cells in HER2-positive and luminal BC are more likely to differentiate into memory B cells, thereby contributing to T cell activation. In contrast, TNBC is characterized by a higher abundance of plasma cells, which arises from diverse T-cell-B cell interactions (168). This study found that T-cell-B cell crosstalk may play a crucial role in shaping the TIME in TNBC, and the associated genes and cell clusters could offer significant prognostic insights for TNBC patients. The antigens released by BC cells are the primary factors driving the proliferation and infiltration of B cells. By alleviating the constraints of the TME on B cells, PTT promotes the differentiation of B cells from a resting state to an activated phenotype over time, which is positively correlated with the expression of genes associated with the overall survival of BC patients (169). Beyond phenotypic changes, these activated B cells also exhibit direct anti-tumor effects. TIL-B can kill cancer cells by recognizing antigens including β-actin and ganglioside GD3, and by acting on the CXCR4/CXCL12 pathway through intercellular contact or via the Fas/FasL pathway (170). Additionally, B cells exert varying effects on other lymphocytes, such as inhibiting the proliferation of CD4+, CD8+, and CD4+CD25− T cells, as well as NK proliferation in response to IL-15 (170).

Currently, there is limited research on the anti-tumor immunotherapy of BC mediated by B cells, with a primary focus on antigen presentation in PTT-mediated immune regulation. Investigating the tumor antigen-specific immune response elicited by tumor-infiltrating B lymphocytes (TIL-B) may enhance our understanding of PTT-mediated BC immunity. IL-10 and Abs may play key roles in tumor immunity mediated by TIL-B. However, TIL-B may also diminish the effectiveness of anti-tumor immunity, potentially promoting tumor growth and metastasis (154). Some studies suggest that this negative regulation could be attributed to the up-regulation of IL-10, TGF-β and PD-L1 in B cells, which inhibits the anti-tumor effects of T cells (171). The antitumor efficacy of T cells may be suppressed by crosstalk or direct interactions between T and B cells mediated by cytokines (172). Furthermore, certain studies indicate that IL-10 and TGF-β may partially influence B cell infiltration in response to PTT or tumor-draining lymph nodes (TDLN), warranting further investigation (173). In addition to T cells, PD-L1 is a critical mediator of regulatory B cells (Bregs) to induce B cell infiltration through the PD-1/PD-L1 axis, highlighting its potential as an anti-tumor therapy (174). PD-L1 inhibits T cell proliferation and T cell-dependent immunogenic chemotherapy, supporting the combined application of PTT and PD-L1 checkpoint inhibitors from a B cell perspective (175, 176). Overall, B cells exhibit both pro-tumor and anti-tumor functions, contingent upon their phenotype, secreted Abs, and the surrounding TME (177). The regulatory mechanisms of BC remain largely unclear, likely due to the heterogeneity of B cell subsets and their functions across different BC types. Understanding these immune mechanisms is critical for identifying novel biomarkers and designing effective therapeutic strategies, thereby necessitating a thorough exploration of the composition and role of B lymphocytes.

#### 3.2.3 Natural killing effect of NK cells

NK cells are cytotoxic lymphocytes that are thought to be early responders against tumors. Earlier studies have highlighted the significance of NK cells in the anti-tumor response in murine models. However, cases of NK cells involvement in human cancer are too rare to thoroughly evaluate their role in cancer. Recent studies have demonstrated that the infiltration of NK cells into tumors is a favorable prognostic factor for non-small cell lung cancer (NSCLC), clear cell renal cell carcinoma (ccRCC), and colon cancer (178–180). In particular, a unique subpopulation of immature NK cells in TME, characterized as Socs3highCD11b-CD27-, has been shown to be associated with the progression and metastasis of TNBC (181). Due to their potent anti-tumor activity and pro-inflammatory effects, NK cells are at the forefront of immunotherapy development. Their anti-tumor effects are primarily mediated through direct cytotoxicity and the activation of other immune cells. Research has shown that NK cells are regulated by a balance of activating and inhibitory receptor signals. This regulatory balance facilitates the release of cytolytic granules containing perforin and granzymes to induce target cell death (127, 128). Additionally, NK cells can also alert the immune system by producing immunomodulatory cytokines (182). Kaili Liu was the first to demonstrate that PTT enhances the cytotoxicity of NK cells. The upregulated genes induced by PTT are positively correlated with favorable clinical outcomes in cancer patients, mainly focusing on the type I and type II interferon pathways (183). PTT significantly enhances the expression of a group of activating effector molecules, including type I/II interferons and NCR1 receptors, thereby promoting NK cell activation. Inflammatory factors such as TNF-α play a crucial role in stimulating NK cells through PTT, establishing a foundation for the positive correlation observed between favorable prognosis and overall survival across several cancer types. Furthermore, IFN-γ cytokines secreted by NK cells contribute to the development of T cell responses in lymph nodes and indirectly influence DCs (184). Localized heating at the tumor site induces a DNA damage response, which subsequently triggers the elevated expression of NKG2D and B7-H6 receptors, characteristic of activated NK cells receptors (185, 186) (**Figure 2B**). Additionally, it has been proposed that PTT enhances T cell-mediated anti-tumor immunity, potentially serving as one of the mechanisms through which PTT activates NK cells in the context of anti-tumor responses. However, tumors counter this immune surveillance by evolving sophisticated evasion mechanisms, particularly through microenvironment-mediated suppression of NK cell recognition.

A prime example of this immune escape strategy is the TME-induced alteration of NK cell ligands, including proteolytic shedding of soluble NKG2D ligands, clinically correlated with poor prognosis (187). In addition, tumor-infiltrating NK cells (TINK) may contribute to tumor development to a certain extent. Influenced by the TME, NK cells release granzymes to activate cancer stem cells via the Wnt signaling pathway. The conversion of NK cells into type 1 ILCs, mediated by the immunosuppressive cytokine TGF-β, has also been identified as a mechanism through which tumors evade NK cells-mediated cytotoxicity within the TME (183). However, PTT has been shown to significantly inhibit the TGF-β-associated signaling pathway, thereby alleviating this immunosuppression. Increasing evidence highlights that the acquisition of immune checkpoint molecules by TINK cells is associated with a regulatory phenotype characterized by T cell inhibition (183). The expression of CTLA4, PD-1, LAG3, and TIGIT has been correlated with NK cells exhaustion across various tumor types (188–191). For example, LAIT has been shown to counteract immunosuppression to some extent by decreasing LAG3 expression (190). Expression of CTLA4 is upregulated to promote immunosuppression (156). Therefore, combining PTT with anti-CTLA4 therapy may enhance the efficacy of cancer treatment. PD-1 is expressed by activated mature NK cells in the lymphoid organs of tumor-bearing hosts, and the PD-1/PD-L1 axis, in turn, regulates the phenotype of NK cells (192). Thus, it may be possible to enhance the immune response mediated by PTT TNBC by improving PD-L1 antibodies through the depletion or inhibition of NK cells, or by alleviating tumor immune suppression with anti-PD-1.

#### 3.2.4 Innate immunity of macrophages

Macrophages are derived from common medullary precursor cells, which play a crucial role in innate immunity. PRR, modulatory receptors, chemokine/cytokine receptors and antigen-presenting molecules on the surface of macrophages facilitate phagocytosis and the elimination of foreign pathogens, including bacteria, fungi and parasites (193). Additionally, various immune factors are released to activate other adaptive immune cells and present foreign antigens to T cells. In different microenvironments, macrophages are polarized into distinct types. M2 tumor-associated macrophages M2 (M2 TAMs) promote the secretion of angiogenic cytokines, which inhibit the proliferation and activation of T cells, thereby dominating the TME in most cancers (194). In BC, especially TNBC, M2 TAMs have been shown to enhance angiogenesis, immunosuppression, and drug resistance, all of which are closely associated with poor prognosis (195, 196). TAMs migrate freely in circulation and bypass the blood-brain barrier, allowing them to accumulate and spread within solid tumors, accounting for approximately 50% of tumor volume in BC (197).

M1 tumor-associated macrophages (M1 TAMs) are typically activated by Toll like receptors (TLRs), which consist mainly of bacterial lipopolysaccharide (LPS), interferon-gamma (IFN-γ), and HSPs (198). It has been found that PTT stimulates the expression of mHSP70 on the surface of MCF-7 cells to increase relatively (113) (**Figure 2B**). HSPs activate M1 TAMs by stimulating TLRs to interact with the TME to generate an immune response (199). In addition, M1 TAMs release pro-inflammatory cytokines, demonstrating phagocytosis and antigen presentation capabilities (200). PTT has been shown to upregulate TNF-α and IL-1/6, enhancing the innate immune response of M1 TAMs (201). Consequently, M1 TAMs may be a pivotal connection point for combining innate and specific immunity. Recent evidence shows that bone metastasis of BC is closely associated with pro-regenerative cytokines produced by M2 TAMs, including TGF-β, VEGF, Arg-1 and IGF-1 (202). Angiogenesis is a critical step in bone regeneration, regulating the osteogenic differentiation of bone marrow mesenchymal stem cells. This mechanistic link suggests that M2 TAMs could be a therapeutic target for bone metastasis in BC. However, NIR laser irradiation not only significantly increases the local temperature surrounding the photothermic agent but also inhibits the expression of transfer-related factors such as matrix metalloproteinases and TGF-β1 (203). Inspired by this, Yu Wei Ge developed a multifunctional CePO_4_/CS/GO scanning sheet that employs PTT to modulate macrophage behavior. This macrophage-targeting strategy enhances angiogenesis and osteogenesis during bone repair, demonstrating therapeutic potential for BC bone metastases. The hypoxic microenvironment of tumors is a key factor inducing the polarization of macrophages into the M2 phenotype, thereby providing nutrients and oxygen to malignant cells and infiltrating host cell populations. In response to hypoxia, factors that promote angiogenesis are produced, which regulate the development of new blood vessels in BC, significantly hindering the efficacy of immunotherapy in tumors (194). It has been proposed that hydrogen peroxide in the TME can redirect pre-tumor M2 TAMs to anti-tumor M1 TAMs (204). In addition, the high concentration of oxygen in the TME inhibits aerobic glycolysis in mitochondria, closely associated with the anti-tumor effects of TAMs activated by LPS and IFN-γ (205). Therefore, reversing hypoxia in the TME is crucial for regulating intracellular gas levels and mitochondrial function, thereby enhancing the effectiveness of PTT-mediated macrophage immunotherapy. For instance, Wang et al. developed a multifunctional Bi/MnPcE4 nanocomposite capable of modulating hypoxic tumor microenvironments. This material promotes macrophage polarization toward anti-tumor subtypes, thereby amplifying PTT-induced immune responses (206).

However, some studies have suggested that the function of TAMs in innate immunity may be inhibited in BC (207). PTT activates TAMs by the TLR receptor pathway, contributing to in tumor immune escape (208). TAMs in rodent models of BC exhibit greater resistance to HSP activation compared to TAMs in normal brain tissue (193). In addition, the Stat3 pathway actively suppresses Th1 mediator while upregulating multiple immunosuppressive factors. This dual regulatory mechanism are considered crucial for tumor immune escape (209). The phenotypes of M1 and M2 TAMs are not fixed; rather, they are influenced by different hormones, cytokines, and apoptotic cells. For instance, IL-4, IL-10, hyaluronic acid, TGF-β, and macrophage colony-stimulating factor (M-CSF) are believed to induce TAMs to differentiate into M2 TAMs in BC (210). The depletion of M2 TAMs is thought to contribute to tumor progression and invasion, thereby elucidating the immune function of TAMs mediated by PTT.

The development of BC is largely attributed to the establishment of complex and dynamic communication between tumor cells and the cells within the TME. A comprehensive understanding of the interactions among various immune cells can enhance our interpretation of the tumor immune effects of PTT from multiple perspectives. IgG secreted by B cells enhances DCs-induced T cell responses or induces CD4+ T cells to differentiate into FoxP3+ Tregs in a TGF-β-dependent manner, thereby inhibiting cytotoxic T cell responses (170). Therefore, future PTT-mediated anti-tumor immunotherapy targeting B cells will emphasize enhancing B cell antigen presentation and inhibiting the TGF-β differentiation of CD4+ T cells. Furthermore, it has been observed that OX40 on T cells may interact with OX40 ligands on B cells, leading to the inhibition of IFN-γ production and CTLs production and/or migration (211). Additionally, PD-L1 expression on B cells plays a significant role in T cell inhibition. Buisseret proposed that PD-L1 expression correlates with a higher density of TILs, including CD19+ B cells, suggesting that B cell infiltration in tumor tissues may be driven by immunotherapy targeting the PD-1/PD-L1 axis (212). NK cells can bind to the Fc region of antibodies produced by B cells to facilitate tumor cell lysis. The presence of NK cells is crucial for the efficacy of TIGIT and PD-L1 checkpoints. Their absence correlates with lower frequencies of TIGIT or TNF-secreting TILs (CD8+) and higher frequencies of PD-1-expressing TILs (CD8+) (191). It has been reported that PD-1 co-expresses with NKG2A in tumor-infiltrating NK cells and CD8+ T cells. *In vitro* and *in vivo* studies have demonstrated that blocking the NKG2A/HLA-E and PD-1/PD-L1 pathways with antibodies results in complete response rates (213, 214). Therefore, the combination of PTT and PD-L1 must consider the impact of NK cells on the PD-1/PD-L1 pathway. The TAMs are believed to arise from the reciprocal communication between BC cells and other cells within the TME (215). TGF-β and IL-10, secreted by M2 TAMs, inhibit CD8+ T cell function by directly repressing the transcription of genes encoding perforin, granzymes, and cytotoxins (216). Additionally, M2 TAMs activate arginase 1 and aminoamide 2,2-dioxygenase, leading to the depletion of arginine and tryptophan in the TME. This metabolic reprogramming impairs the proliferation and survival of T and NK cells (217). In particular, it is widely reported that polymorphonuclear myeloid-derived suppressor cells (PMN-MDSCs) contribute to the depletion of ARG1 in the TME by releasing nitric oxide and arginase, thereby inhibiting T cell function (218). A recent study indicates that T cells and NK cells have complementary roles in tumor immunity, offering opportunities to enhance the efficacy of PTT. The experiments demonstrated that NK cells recruit DCs to the tumor and provide a niche supporting their survival, thereby enhancing the ability of CD8+ T cells to respond. Meanwhile, IL-2 secreted by T cells can activate the anti-tumor function of NK cells (219) (**Figure 2**).

## 4 Clinical research

The high development costs of PTAs have limited the translation of PTT preclinical research into clinical studies. In preclinical tumor models, PTT reliant on PTAs often enables rapid and reproducible tumor ablation. However, clinical research has primarily focused on developing integrated laser device ablation systems that do not depend on exogenous PTAs. In fact, PTT treatment for cancer patients can also be provided using laser devices alone. For example, Nd: YAG laser endoscopic photocoagulation has been utilized to ablate obstructive bronchial cancer, thereby achieving therapeutic effects (**Table 1**). Additionally, laser interstitial thermal therapy (LITT), which typically involves the placement of laser fibers into tumors, has been applied to various solid tumors, such as prostate and liver tumors (224, 225). Some LITT devices have successfully advanced to the later stages of clinical trials. Among them, the Novilase system provides a LITT method for fibroadenoma and is currently undergoing a Phase III trial (NCT00807924). In a Phase II trial of percutaneous laser ablation (PLA) for the treatment of invasive ductal BC, complete tumor ablation was achieved in 51 out of 61 patients (84%). The MRI negative predictive value (NPV) for all patients was 92.2% (95% confidence interval [CI], 71.9–91.9%) (220) (**Table 1**).

**Table 1:** Clinical research of BC in the treatment of PTT.

Cancer	Laser	Method	Results	Reference
IDC	PLA	Patients by pre-ablation MRI were treated with PLA, and underwent a 28-day post-ablation MRI.	84% patients had complete tumour ablation	(220)
metastatic BC	LIT	ICG for selective thermal effect, and immunoadjuvant (glycated chitosan) for immunological stimulation	the objective response rate was 62.5% and the clinical beneficial response rate was 75%.	(221)
breast fibroadenomas	Nd: YAG	Under real-time ultrasound monitoring, Nd: YAG laser (1,064 nm wavelength) was used at 2 W for 300 sec (600 J) in a continuous wave mode to produce interstitial hyperthermia.	There was significant decrease in clinical and sonographic sizes.	(222)
small benign breast lesions	LAT	US-guided laser ablation was performed on 19 benign breast lesions in 10 patients.	hypoechoic lesions having inner hyperechogenicity with or without a hypoechoic center.	(223)

Compared to traditional LITT, PTT centered on PTA offers superior selectivity and enables treatment at lower power levels. However, to date, clinical research on PTA-enhanced PTT has been limited to a few early-stage trials. Most of these studies have focused on other types of tumors, such as prostate cancer, with only a small number reporting clinical trials for BC. For instance, Xiaosong Li et al. documented the clinical translation of localized PTT using ICG in patients with refractory advanced metastatic BC (221) (**Table 1**). ICG absorbed by tumor cells, when irradiated by an 805 nm laser with a power of 1W/cm², causes the tumor cells to expand and dissolve, thereby releasing antigens. The immune system is activated through the immunostimulation of the immune adjuvant glycated chitosan, to achieve an effective and protective immune response against residual tumor cells. Among the eight evaluable patients, the clinical benefit response rate was 75%, with no serious adverse events reported (221) (**Table 1**). Clinical translational results indicate that PTT holds significant potential in the treatment of metastatic BC.

Despite optimistic preclinical studies, the clinical translation of PTT faces multiple challenges. Since the breast is primarily composed of adipose tissue, traditional NIR-I (750–1000 nm) lasers have insufficient penetration (226). Due to greater penetration depth, lower energy dissipation, and minimal toxicity to normal tissues, the second region (NIR-II, 1000–1500 nm) demonstrates significant superiority. In fact, the clinical conditions of each individual’s breast vary, and the chosen wavelength may also differ. Moreover, there are differences in light absorption and penetration among different tissue types (227). Additionally, the prolonged retention of nanomaterials (such as gold and black phosphorus) may induce inflammation or fibrosis, necessitating the development of degradable materials (such as PLGA encapsulation systems). In fact, BC is characterized by high heterogeneity, which is also one of the key factors limiting the clinical translation of PTT technology. Different subtypes of BC exhibit distinct clinical characteristics, requiring the design of subtype-specific targeting strategies.

## 5 Challenges and potential problems

It is undeniable that immune cells are crucial to the progression, invasion, and metastasis of BC. As protectors within the body, immune cells actively target cancer cells while safeguarding normal cells. PTT serves as a mechanism that can activate and eliminate tumor systems, providing opportunities to dismantle tumor structures and stimulate the immune response. Although PTT can release antigens to activate the immune system, it presents several challenges and potential issues that must be addressed.

Local temperature of tumor is an important factor affecting tumor immune stress, which is largely limited by the penetration depth of NIR. Hyperthermic temperatures (above 50 °C) induce the high expression of macrophage polarization factor, leading to the evolution of the M2 phenotype (49). In contrast, PCD induced by lower temperature (42-45°C) avoids inflammation caused by the leakage of cytoplasmic contents and effectively stimulates anti-tumor immune responses. Furthermore, DNA damage generated through PTT promotes high expression of HSPs to facilitate M1 polarization. However, HSPs are also responsible for cellular thermal tolerance, reducing photothermal efficacy. Recent studies have shown that the location of PTT also affects its anti-tumor effect. When used in the extracellular space, the converted heat will rapidly diffuse and prevent the accumulation required for effective cytotoxic effects. When used on cell membranes, its low thermal conductivity hinders the rapid diffusion of heat, resulting in a higher temperature gradient and more severe membrane damage. Therefore, compared with extracellular locations, PTT targeting the cell membrane is more effective (122). When used on cell membranes, its low thermal conductivity hinders the rapid diffusion of heat, resulting in a higher temperature gradient and more severe membrane damage. Therefore, compared with the extracellular location, PTT targeting the cell membrane is more direct. In addition, the PCD with PTT is influenced by various signaling pathways, including STINA, Beclin-1, PI3K/AKT/mTOR, CMA, and TRPV1. Notably, dying tumor cells may not release sufficient antigens or generate significant immune stress, complicating the design of anti-tumor immunotherapies utilizing PTT. These challenges must be prioritized when developing NPs for PTT.

Immunotherapy strategies with PTT primarily aim to induce tumor cell death, thereby releasing antigens and activating various immune cells. However, the immune system is a highly coordinated system, which will upset the balance with unpredictable adverse effects. For example, PTT promotes the secretion of IFNs and TNF-α by DCs, leading to some changes associated with T cells, macrophages, and NK cells. Furthermore, PTT influences T and B cells primarily through cytokine signaling, which may be insufficient for effective treatment. Consequently, PTT-based immunotherapies necessitate the incorporation of additional pharmacological agents, raising the complex issue of how to precisely regulate photothermal-induced immune enhancement rather than suppression. Additionally, the anti-tumor immune response must account for the distinct spatial and temporal distribution of immune cells. It is also essential to consider the impacts of immune cell phenotypes, secreted Abs, and the TME on the PTT-mediated immune response (177). Therefore, strategies for anti-tumor immunomodulation involving PTT should be meticulously developed and assessed. In contrast to T cell-mediated immune strategies, tumor antigen-specific immune responses initiated by TIL-B have been demonstrated to play a role in regulating PTT-mediated BC immunity. While B cells present antigens to T cells, the process of antibody-dependent cell-mediated cytotoxicity (ADCC) leads to tumor cell lysis. Unfortunately, research in this area remains extremely limited, likely due to the largely unknown regulatory mechanisms governing BC.

However, some studies have found that the immune signal induced by PTT is too weak to fully activate the immune system (228). In addition, some immunosuppressive factors in the TME can lead to frequent recurrence and metastasis of tumors after PTT (229). At present, in order to enhance and regulate the immune response, the combined strategy tends to regulate the immune microenvironment. For instance, some cutting-edge studies have shown that divalent manganese ions (Mn^2+^) can act as innate immune adjuvants to enhance the immune signaling of PTT. Studies have shown that Mn^2+^ promotes the maturation/activation of DCs and enhances the presentation of DCs antigens and the differentiation/activation of CD8+ T cells through the cyclic GMP-AMP synthase-stimulating factor interferon gene (cGAS-STING) pathway (230). However, the added Mn^2+^ can lead to insufficient accumulation of Mn^2+^ in the TME, which may be toxic to normal tissues. Currently, combined strategies tend to modulate the immune microenvironment. However, the immune microenvironment in BC is a complex system, and our understanding of its components and intricate interactions remains insufficient. Consequently, even when different combination strategies are employed, selecting a tailored solution remains problematic. In addition, most NPs-based PTT immune checkpoint inhibitors encounter similar challenges in determining the appropriate regimen for specific immune checkpoints based on PTT. The specific analysis of different immune checkpoints in relation to the immune response to PTT does not appear to have been adequately addressed. Additionally, it is essential to consider the autoimmune diseases that may arise from the use of immune checkpoint inhibitors (231). It is important to note that most studies are confined to animal models, as human tumors are heterogeneous and characterized by numerous mutations. TME varies from patient to patient, and strategies that demonstrate promising treatment results in animal models may not translate effectively to humans. Therefore, to enhance the design and evaluation of nano-delivery systems, it is imperative to establish animal models that more closely resemble human breast tumors.

## 6 Conclusions and outlook

In recent years, there have been relatively few studies on PTT of tumors, and the existing research has not yet demonstrated impressive efficacy. However, PTT, which is based on the regulation of immune cells, offers a glimmer of hope for the treatment of BC. This article reviews the immunomodulatory mechanisms of PTT in BC, including antigen release, antigen presentation and activation of immune cells. In summary, several limitations of PTT must still be addressed, such as heat resistance, low immunogenicity, constraints of the TME, and potential therapeutic risks. Nevertheless, PTT remains a promising avenue for research aimed at improving tumor treatment outcomes. Furthermore, the immune system is a complex and integrated network, and PTT may simultaneously target various immune cells and cytokines. Ultimately, we expect that ongoing efforts to advance the development of new PTT-based immunotherapy approaches and strategies to target and reshape the TME will lead to more effective direct or adjunctive treatments for BC.

## Glossary

BC: Breast cancer
PTT: Photothermal therapy
PTAs: photothermal agents
APCs: antigen-presenting cells
HER2: human epidermal growth factor receptor 2
HR: hormone receptor
HSPs: Heat shock proteins
EMT: epithelial-mesenchymal transformation
BCSCs: breast cancer stem cells
NIR: Near infrared
MTX: Methotrexate
ECM: the extracellular matrix
PCD: programmed cell death
VEGF: vascular endothelial growth factor
MMP: metalloproteinases
TGF-β1: transforming growth factor-β1
ICD: immunogenic cell death
DCs: dendritic cells
NK: natural killer
IBC: inflammatory breast cancer
DR: the death domain receptor
TNF-1α: Tumor necrosis factor-1α
ATP: adenosine triphosphate
RIP3: receptor-interacting protein 3
MLKL: mixed lineage kinase domain-like protein
TRAIL: TNF-related apoptosis inducing ligand
ER: the endoplasmic reticulum
Cyt c: cytochrome c
Apaf-1: apoptotic protease activating factor-1
Bcl-2: B-cell lymphoma-2
NP: nanoparticle
PPTT: plasma-nanoparticle-mediated photothermal therapy
KMTs: Lysine methyltransferase
KDMs: Lysine demethylase
KDM3A: Lysine Demethylase 3A
CMA: chaperone-mediated autophagy
LC3: Light chain 3
Vps34: vacuolar protein sorting 34
TNBC: triple-negative breast cancer
HIF-1: hypoxia-inducible factor 1
ROS: reactive oxygen species
TSC1/2: tuberous sclerosis 1/2
PI3K: the phosphoinositide 3-kinase
AKT: protein kinase B
mTOR: mechanistic target of rapamycin
Atg5: autophagy associated gene 5
CQ: chloroquine
Gox: glucose oxidase
MHC-I: major histocompatibility complex Class I
CS: the chaperone system
UPS: the ubiquitin-proteasome system
TME: tumor microenvironment
DAMPs: damage-associated molecular patterns
GSDME: the activation of Gasdermin E
HMGB1: High mobility group box 1
CRT: Calreticulin
TRPV1: the transient receptor potential vanilloid 1
DHFR: dihydrofolate reductase
mHsp70: mitochondrial HSP70
TAAs: tumor-associated antigens
PRRs: pattern recognition receptors
cDCs: conventional dendritic cells
pDCs: plasmacytoid dendritic cells
PAMPs: pathogen-associated molecular patterns
TLR: Toll-like receptor
Abs: antibodies
TDPAs: tumor-derived peptide antigens
ACNs: antigen-trapping nanoparticles
GC: glycated chitosan
ICB: immune checkpoint blockade
PD-L1: programmed cell death ligand 1
IgG: immunoglobulin G
TCR: T cell antigen recognition receptor
Tregs: regulatory T cells
CAFs: cancer-associated fibroblasts
TILs: Tumor-infiltrating lymphocytes
TIL-B: tumor-infiltrating B lymphocytes
TDLN: tumor-draining lymph nodes
Bregs: regulatory B cells
NSCLC: non-small cell lung cancer
ccRCC: clear cell renal cell carcinoma
TINK: tumor-infiltrating NK cells
M2 TAMs: M2 tumor-associated macrophages M2
M1 TAMs: M1 tumor-associated macrophages
LPS: lipopolysaccharide
IFN-γ: interferon-gamma
M-CSF: macrophage colony-stimulating factor
TIL-B: tumor-infiltrating B cells
CTLs: cytotoxic T lymphocytes
TLRs: Toll like receptors
HMGB1: high mobility group protein 1
ICG: Indocyanine green
ICD: immune cell death
